# A Review on Health Benefits of *Malva sylvestris* L. Nutritional Compounds for Metabolites, Antioxidants, and Anti-Inflammatory, Anticancer, and Antimicrobial Applications

**DOI:** 10.1155/2021/5548404

**Published:** 2021-08-14

**Authors:** Seyyed Mojtaba Mousavi, Seyyed Alireza Hashemi, Gity Behbudi, Sargol Mazraedoost, Navid Omidifar, Ahmad Gholami, Wei-Hung Chiang, Aziz Babapoor, Nelson Pynadathu Rumjit

**Affiliations:** ^1^Department of Chemical Engineering, National Taiwan University of Science and Technology, Taipei, Taiwan; ^2^Department of Mechanical Engineering, Center for Nanofibers and Nanotechnology, National University of Singapore, Singapore; ^3^Department of Chemical Engineering, University of Mohaghegh Ardabili, Ardabil, Iran; ^4^Biotechnology Research Center, Shiraz University of Medical Sciences, Shiraz, Iran; ^5^Department of Pathology, Shiraz University of Medical Sciences, Shiraz, Iran; ^6^Pharmaceutical Sciences Research Center, Shiraz University of Medical Sciences, Shiraz, Iran; ^7^Nanotechnology and Catalysis Research Centre (NANOCAT), Institute for Advanced Studies (IAS), University of Malaya (UM), Kuala Lumpur, Malaysia

## Abstract

The utilization of medicinal plants and their derivatives in treating illnesses is more appropriately recognized as herbal remedy than traditional medicine. For centuries, medicinal herbs have been used for the treatment of diseases in many countries. *Malva sylvestris* L. is a kind of mallow derived from Malvaceae species and is recognized as common mallow. This amazing plant has antimicrobial, hepatoprotective, anti-inflammatory, and antioxidant properties and is considered as one of the most promising herbal medicinal species. This plant's traditional use in treating many diseases and preparing pharmaceutical compounds can show us how to know in depth the plant origin of drugs used to produce antibiotics and other therapeutic agents.

## 1. Introduction

**Figure 1:**
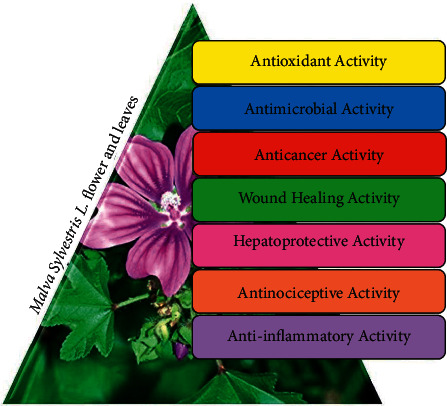
Flower of *M. sylvestris* and different biological activities [7].

*Malva sylvestris* L. (*M. sylvestris*) is one of the medicinal plants commonly recognized as common mallow in Europe, Iran, Pakistan, and India. *M. sylvestris* is a biennial-perennial herbaceous plant commonly found in North Africa, Europe, and Southwest Asia [1, 2]. The plant generally grows in moist areas, for instance, near marshes, ditches, oceans, riverbanks, and meadows [3]. Due to the softening properties of this plant, the Romans and ancient Greeks used it as a softener [4, 5]. Traditionally, these medicinal plants have been used to treat several infections and diseases, such as cold, burn, cough, tonsillitis, bronchitis, digestive problems, eczema, and cut wounds under different weather conditions [6]. As a natural product, *M. sylvestris* leaves and flowers showed various therapeutic effects.  Figure 1 shows some of the medicinal applications of this plant.

Fluid extracts of *M. sylvestris* leaves and flowers are used to treat inflammatory diseases of mucous membranes, cystitis, and diarrhea [8]. This plant derives its restoration competencies from the mucilage and flavonoids located in the vegetation and leaves. Young leaves, shoots, flowers, and fruits are consumed in salads, soups, or boiled root vegetables. Flowering flora in the mallow family (Malvaceae) or hibiscus generally include the *Malva* and *Hibiscus* species. *Hibiscus* species comprise the swamp-rose mallow (*Hibiscus moscheutos*); another type of rose mallow (*Hibiscus militaris*), a shrub that grows to a peak of 2 m; and superb rose mallow (*Hibiscus grandiflorus*), with ample white to purplish flowers. Herbal medicine is one of the oldest treatment practices followed by humans. In the last 30 years, medicine specialists focus on the use of medicinal herbs in preventing and treating diseases. Among the numerous species used in traditional medicine, Malvaceae family is more prominent due to its diverse applications, and its consumption can be traced back to 3000 years ago.

The marshmallow (*Althea officinalis*), generally found in swamplands or marshes near the sea, is indigenous to North America and local to Europe and North Africa. Recently, its root has been used to make sweets. *Malva* plant in India, with a maximum height of up to 40 cm, is prescribed for the treatment of cough and cold due to respiratory problems involved and for the treatment of gastrointestinal problems [9]. This drug is used in Brazil to treat bronchitis, wounds, colitis, and hemorrhoids [10]. The chemicals in the leaf of *Malva*, which has many vitamins, allow for faster recovery by secreting certain analgesics to reduce pain and discomfort [11].

**Figure 2:**
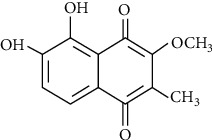
Chemical framework of malvone A, a phytoalexin found in *M. sylvestris* [7].

Medicinal plants have been frequently used to treat a variety of human diseases. Over the last century, the use of vegetation in medication, hematology, oncology, and immunology has affected the identity of natural composites: codeine, taxol, vinblastine, morphine, and cocaine, among others. The results of several studies have shown that *Malva* extract contains different compounds, including phenolic derivatives, flavonoids, terpenoids, catalase enzymes, sulfite oxidase, fatty acids, and certain strolls (specifically essential fatty acids such as omega-3 and omega-6), beta carotene, and vitamins C and E, which have anti-inflammatory and antioxidant properties [12–15]. Therefore, it can protect the kidney against injuries due to renal toxicity resulting from the cisplatin and vanadium system [16]. Extensive research shows that this plant, with different chemical compounds, can minimize liver damage caused by carbon tetrachloride. *M. sylvestris* has antimicrobial, antinociceptive, hepatoprotective, wound-healing, anticancer, anti-inflammatory, and potent antioxidant properties (Figure 2). Also, this plant contains many valuable compounds such as strong antioxidants and carbohydrates and unsaturated fatty acids. Tannins, flavonoids, phenolic compounds, and ascorbic acid found in the *Malva* plant are used to treat most cancers and for wound-healing [2, 7, 9, 17].

### 1.1. Phytochemistry

The prevalence of using plant antioxidants, considering their use in various research and applied aspects of antioxidants, especially the valuable compounds underlying phenolic induction with its groups with free radical absorption, plays an essential role in spreading its use as an oxidation preventive agent [18]. In the study conducted by Nawwar et al. [19], the phenol carboxylic and free organic acids were methylated. By using the following formula, the contents of components were calculated:

(1) $$\begin{array}{c} C\left(\mathrm{mg}/\mathrm{kg}\right)=K_{1}\times K_{2}\times 1000, \end{array}$$

where *K*_1_=*A*_1_/*A*_2_ (*A*_1_ and *A*_2_ are the peak areas of the test and standard compounds, respectively) and *K*_2_ is the mass of the internal standard (*μ*g) added to the sample. The component combination of organic acids is shown in Table 1.

A total of 13 organic acids extracted from the leaves of *M. sylvestris* are known, including malonate (1284.4 mg/kg), malate (3510.0 mg/kg), oxalate (4170.7 mg/kg), fumarate (6924.8 mg/kg), and citrate (13133.2 mg/kg). These compounds contribute to developing the immunostimulant and antioxidant properties for *M. sylvestris* and their preparations based on these natural compounds [5, 20]. It is proven that these flavonoids structures, along with other phenolic compounds, are present in higher amounts in the *M. sylvestris* flowers and have more effective antioxidant properties, as given in Tables 1 and 2. The antioxidant property was found to be more profound in flower extracts of *M. sylvestris* based on the results of the (2,2-diphenyl-1-picryl-hydrazyl-hydrate) free radical method (DPPH assay) and ferric reducing antioxidant power assay (FRAP assay). The researchers further discovered more flavonoids and phenolic contents and antioxidants in leaves than in leafy flower stems and flowers when 95% ethanol was removed [21].

**Figure 3:**
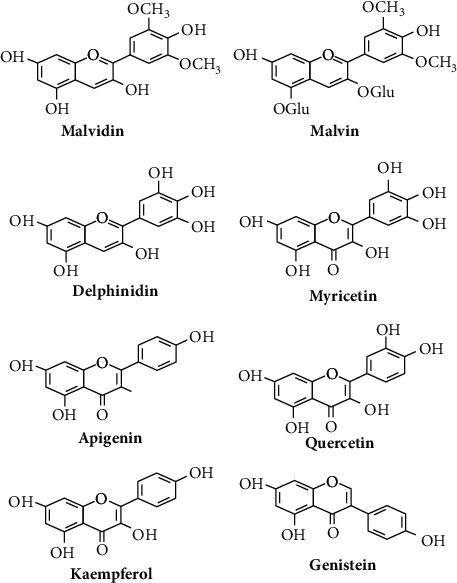
Some flavonoids of *M. sylvestris* [7].

A major phytoalexin found in *M. sylvestris* was 2-methyl-3-methoxy-5,6-dihydroxy-1,4-naphthoquinone, known as malvone (Figure 2).  Figure 3 shows some flavonoids that have a significant therapeutic effect.

### 1.2. Carbohydrate Content in *M. sylvestris*

Research has shown that most carbohydrates in plant materials derived from polysaccharides demonstrate an unknown mechanism during antioxidant activity. In animal experiments, these polysaccharides, especially pectins, are mainly found in plant tissues, show antioxidant and antidiabetic properties, and even adjust blood insulin, as given in Table 3.

The leaves are the richest in flavonoids, and this explains their therapeutic properties in traditional medicine.

### 1.3. Mucilages

The mucilages contain trehalose, galactose, sucrose, glucose, fructose, mannose, rhamnose, galacturonic, and glucuronic acid, but 2″-O-a-(4-O-methyl-a-d-glucuronosyl)-xylotriose, raffinose, fucose, xylose, arabinose, and uronic acid have also been found in *M. sylvestris*. It is considered an essential antimicrobial agent due to its resistance to the pathogen *Verticillium dahliae* [22].

### 1.4. Pigments

Qualitative analysis of acetone extracts from *M. sylvestris* has been done using chromatography. These assessments approve the presence of xanthophylls, chlorophyll B, and chlorophyll A [23].

### 1.5. Fatty Acids/Sterols

In *M. sylvestris* leaves, the presence of the stigmasterol, g-sitosterol, and the steroid campesterol has been reported [22]. The plant growth status affects the qualitative and quantitative constituents of these materials. Lipids exist separately in the flowering stems, immature fruits, flowers, and leaves [2]. These include tricosanoic acid, heneicosanoic acid (C20:3n3 + C21:0), lignoceric acid, 14-eicosadienoic acid, cis-11, behenic acid, arachidic acid, eicosenoic acid, a-linolenic acid, linoleic acid, heptadecanoic acid, palmitoleic acid, pentadecanoic acid, oleic acid, stearic acid, myristic acid, palmitic acid, myristoleic acid, lauric acid, capric acid, caprylic acid, and caproic acid. Extracts from leaf upon rapid cure with methanol and acetyl chloride contain 0.47% lipids and linolenic acid (42.21%). Because of the availability of indispensable fatty acids such as omega-3 and omega-6, *M. sylvestris* plays a pivotal role as a nutraceutical food. The consumption of omega-3 fatty acid compounds can prevent many diseases, such as coronary artery disease, diabetes, and cancer.

### 1.6. Chemical Elements

Assessment of the leaves of *M. sylvestris* has shown the presence of essential and nonessential metallic elements, halogens, and nonmetals. Analysis was performed using plasma optical emission spectrometry (ICP-OES), and the presence of Zr, Zn, U, Tl, Sr, Pb, Ni, Na, Mn, Mg, Sn, La, K, Si, Fe, Cu, Cr, Co, Ca, Bi, Ba, B, and Al was also shown [24]. *M. sylvestris* has exhibited a considerable ability to accumulate substantial metals (Zn, Pb, Ni, Cu, and Cd) from soils rich in these materials. Thus, it is crucial to address this issue in affected populations living in hazardous zones [25].

### 1.7. Vitamins

One of the natural properties of *M. sylvestris* is the human cell supplementation using ascorbic acids (vitamin C) and tocopherols (vitamin E). Vitamin E is considered a remarkable cancer prevention agent of the tocopherols in the human body [2, 26].

### 1.8. Enzymes

**Figure 4:**
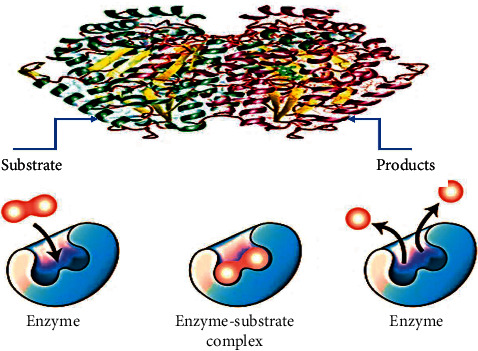
Mechanism of enzyme activity.

In the oxidative degradation of sulfur-containing amino acids, sulfite oxidase as an enzyme plays an integral role in ending the reaction (Figure 4). The absence of this enzyme might lead to death. Sulfite oxidase has additionally been discovered in the leaves of *M. sylvestris* and has been found in numerous bacteria and animal species [26–29]. Various phenolic derivatives have been found in extracts from different parts of *M. sylvestris* [26, 27].

### 1.9. Pharmacological Activity

*M. sylvestris* has been reported for use in the therapy of oral diseases. Anti-inflammatory and antimicrobial effects on the antimicrobial outcomes of ethanolic extracts from *M. sylvestris* stems were investigated in contrast to methicillin-resistant *Staphylococcus aureus* through biofilm adherence/formation tests and planktonic growth [30].

The biofilm foundation method showed that ethanolic stem extracts had medium activity in planktonic growth tests against *S. aureus* with bounded bacteriostatic effects [30–32]. Ethanolic extracts obtained from the inflorescences and leaves of *M. sylvestris* have a significant impact on *Helicobacter pylori*. This bacterial strain plays an essential role in treating peptic ulcers and gastric cancers [33].

Hence, in the agar, diameter inhibition zones, crude methanolic extracts did not significantly inhibit strains of *Saccharomyces cerevisiae*, *Bordetella bronchiseptica*, *Candida albicans*, *Serratia marcescens*, *B. pumilus*, *B. cereus, B. subtilis*, *M. luteus*, *S. epidermidis*, *K. pneumonia*, *S. aureus*, *E. coli*, *P. fluorescens*, and *P. aeruginosa* [34–36]. The useability of *M. sylvestris* in mice considering the aqueous extract as an anti-inflammatory agent has been investigated. Research has shown that this type of extract might significantly reduce inflammation. The hydroalcoholic extract received from *M. sylvestris* leaves exhibited an anti-inflammation effect on croton oil-induced swelling in the ears of mice. The extract outcome has been proven by these facts [37].

The pharmacological activities of *M. sylvestris* are summarized in Table 4. Claimed patents are listed in Table 5. Other details related to the medicinal use of *M. sylvestris* are given in Table 6.

### 1.10. Traditional Uses of *Malva* Species

The traditional-ethnobotanical uses of *M. sylvestris* are given in Table 7.

Gas chromatography and mass spectroscopy analyses were carried out on compounds found in methanolic leaf extracts of *M. sylvestris*; results are shown in Table 8 [7].

### 1.11. Nutritional Values of Different Parts of the Plant

The investigation of the dietary arrangement of each one of those parts is necessary. The plant pieces and corresponding nutritional values are given in Table 9. Besides, Table 10 summarizes the sugar content in different parts of *M. sylvestris*.

Gas chromatography-mass spectrometry (GC-MS) evaluation is a practical approach used for countless functions with the most excellent sensitivity and specificity. A volume of 1 *μ*L methanol extract of *Malva sylvestris* was infused into the GC-MS and inspected typically for 45 minutes. The period since the infusion was made (initial time) to when washing occurred is referred to as the retention time (RT) [81, 82]. Helium fuel containing an eluent was used as a carrier [83].

### 1.12. Antioxidant Activity

*M. sylvestris* has antiradical properties due to high phenolic contents and is capable of preventing oxidation. Flavenoid compounds in this plant have high inhibitory power. These plants are also free of complications in comparison to chemical drugs [84]. The production of different oxygen species over the body's antioxidants causes oxidative stress. Evidence suggests that stress is one of the essential factors of aging in brain function, liver disease, cardiovascular disorders, and cancer [85].

### 1.13. Anti-Inflammatory Activity

Several research groups have investigated *M. sylvestris* anti-inflammatory activity [35]. Their results support the notion that the compound malvidin 3-glucoside seems to be primarily accountable for this effect, and *M. sylvestris* leaves possess topical anti-inflammatory properties. The results of studies on the antimicrobial properties of *M. sylvestris* indicate that the plant also has antibacterial and antiviral activity against many human pathogens [86].

### 1.14. Anticancer Activity

**Figure 5:**
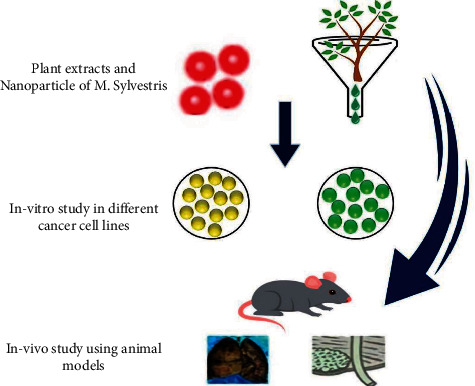
*In vivo* and *in vitro* anticancer activities of *M. sylvestris* against different types of cancer [41].

Cancer is a generic term for a significant group of diseases that can affect any part of the body. Based on the report of World Health Organization (WHO), cancer is a leading cause of death universally. Reports show that *M. sylvestris* possesses anticancer properties. Daniela et al. [41] demonstrated cytotoxic activity of *M. sylvestris* leaf extracts on murine using an MTT assay and human cancer cell lines. The biological test found that *M. sylvestris* extracts significantly decrease cancer cell lines (Figure 5) [5, 41, 87, 88].

### 1.15. Wound Healing Activity

The topical application of the ethanolic hydroalcoholic extract of *Malva* leaves in a dose-dependent manner increases the rate of contraction of skin ulcers and reduces the duration of its repair process in rats. On the other hand, fiber plants are responsible for producing and secreting collagen. Protein collagens are a central extracellular matrix, which leads to an increase in the ability of wound edges to bind to each other.

### 1.16. Hepatoprotective Activity

The liver should be physiologically involved in all vital functions of the body. Any malfunction in the liver causes a set of disorders that can cause irreparable damage to this member; influential factors such as oxidative stress, free radicals, chemicals, viruses, and medicines can cause liver tissue degradation [89–91]. The literature confirmed the presence of antioxidant compounds in *M. sylvestris*. These compounds, in turn, remove the free radicals and help protect tissues, especially in the liver [92].

### 1.17. Antiosteoporosis Activity

Because of the imbalance between osteoblast and osteoclast activities, osteoporosis leads to weakening bone strength and elevation of fracture risk [93, 94]. *M. sylvestris* aqueous extracts can induce the activity of the signaling pathways and affect the osteoblast in an osteoclast difference [12, 86].

### 1.18. Antinociceptive Activity

The antinociceptive activity of *M. sylvestris* aqueous extracts was assessed against traditional pain models in mice by Esteves et al. [10]. Extensive antinociceptive activity was demonstrated in the writhing test (76.4% of inhibition), as well as inhibition of inflammation (46.6%) and neurogenic (61.8%) phases of the formalin model. Their outcomes suggest that *M. sylvestris* possesses stimulating substances, which act as antinociceptive agents.

### 1.19. Antimicrobial Activity

*M. sylvestris* performs antimicrobial activities against various bacterial and fungal species. The disc diffusion method has reported the antimicrobial activity of *M. sylvestris* extracts against different bacterial species. The researchers found that *M. sylvestris* has moderate activity against selected microorganisms associated with typical antibiotics [95].

De Souza et al. [96] studied the antimicrobial activity of *M. sylvestris* aerial part extracts against *C. Albicans, S. aureus, M. luteus, Bacillus subtilis, S. epidermidis, E. coli,* and *S. cerevisiae* [97]. Their study reported that ethanol extracts of *M. sylvestris* were active against *P. aeruginosa*, *B. subtilis*, and *E. coli*, whereas methanol extracts showed activity only against *S. cerevisiae* [98]. Their results demonstrated that *M. sylvestris* extracts inhibited the *in vitro* microbial activity. Other studies showed that the seed oil inhibits the growth of all microorganisms tested except the Gram-negative bacteria *P. aeruginosa* [99–101].

### 1.20. Preventive Effect of *M. sylvestris* on Urinary Toxicity after Radiation Therapy in Prostate Cancer

*M. sylvestris* has a preventive effect on urinary toxicity after radiation therapy in prostate cancer in terms of relieving the pain related to external beam radiation therapy- (EBRT-) induced urinary toxicity. Up-to-date radiotherapy techniques, for instance, three-dimensional conformal radiation therapy (3D-CRT) and intensity-modulated radiation therapy (IMRT), can reduce genitourinary and gastrointestinal toxicity induced by EBRT [102].

### 1.21. Antifungal Assay

The antifungal activities of the plant extracts were the same against *Penicillium* spp., *C. Albicans*, *Aspergillus niger*, *Candida kefir*, and *Sclerotinia sclerotiorum* by the circle dissemination technique. Amphotericin B (10 *µ*g) was considered a positive control, and the plates were cultured at 30°C for 48 hours. The minimal inhibitory concentrations (MICs) of the concentrates against the test microorganisms were controlled by the agar diffusion strategy [3, 103, 104].

### 1.22. Healing of Atopic Dermatitis

*M. sylvestris* is the most common dermatological ailment treatment, for example, atopic dermatitis; however, conventional therapeutics, such as corticosteroids and antihistamines, have no effects [105]. Natural agents, which generally have no extensive side effects, could be used to determine its efficacy. In this study, its effectiveness in treating atopic dermatitis was assessed and it could topically be used as an effective cream to reduce the dermatitis symptoms in children.

## 2. Conclusion

This review showed the significance of *M. sylvestris* as a medicinal herb and functional food. Findings indicate that relatively extensive research has been carried out on chemical compounds and pharmacological effects, as well as different aspects of the *Malva* plant. *M. sylvestris* is an important resourceful plant because of its effective medicinal properties. Studies have proven its potential for health benefits due to its antioxidant activity, anti-inflammatory activity, anticancer activity, wound-healing activity, hepatoprotective activity, antinociceptive activity, and antimicrobial activity. The leaves, flowers, and roots are used for medicinal reasons. Herein, one-of-its-kind organic activities of *M. sylvestris* L., traditional uses, main phytochemical compounds detected in methanolic extracts, and pharmacological activities of *M. sylvestris* were reviewed.

## Figures and Tables

**Table 1:** Quantitative contents of organic acids in leaves of *M. sylvestris* [20].

Acid	Retention time (min)	Content (mg/kg)
Oxalic	8.88	4170.7
Malonic	11.13	1284.4
Fumaric	11.97	6924.8
Succinic	12.95	644.9
Benzoic	13.96	60.1
Glutaric	15.51	37.7
Phenylacetic	16.62	103.6
Salicylic	16.93	219.0
Malic	21.32	3510.0
Citric	28.46	13133.2
Vanillic	31.33	84.3
Ferulic	38.99	397.7
*p*-Coumaric	39.73	65.9

**Table 2:** *In vitro* antioxidant activity of *M. sylvestris* flowers and leaves, complete flavenoid content, and total phenolic content (TPC) [21].

Sample	TPC (mg GAE/g FW)	Total flavonoids (mg QE/g FW)	FRAP	DPPH
			mM TE/g FW	
Mallow leaves	1.42 ± 0.14	0.76 ± 0.19	4.04 ± 0.85	3.88 ± 0.51
Mallow flowers	6.32 ± 0.13	1.45 ± 0.21	6.01 ± 0.54	5.98 ± 0.43

**Table 3:** *In vitro* antioxidant activity, total flavenoid content, and total phenolic content of *M. sylvestris* leaves and flowers [21].

Sample	Fructose	Glucose	Sucrose	Reducing sugars	Total soluble carbohydrates
Mallow leaves	0.88	0.61	0.46	2.1	42.9
Mallow flowers	2.03	0.93	0.21	5.5	47.0

**Table 4:** *M. sylvestris* pharmacological activities.

Activity	Models	Extract/pharmaceutical preparations	Findings
Biochemical profile	Extract intake in rats by drinking water	Aqueous extract from aerial parts	Bodyweight dosages (400 and 800 mg/kg) resulted in a significant rise in serum triglycerides, while other lipids, liver enzyme parameters, and glycaemic (alanine and aspartate transaminases, alkaline phosphatase, lactate dehydrogenase) were unaffected [35]

Bioadhesive mucous membranes	*Ex vivo* system (mucous membranes prepared buccal region tissue from killed pigs)	Aqueous extracts (flowers)	Less bioadhesion for epithelial tissue. Not feasible to correlate rehydration effects in this study, anti-irritative and anti-inflammatory [38]

Antiaging	Quantitative reverse transcriptase-PCR (polymerase chain reaction) and DNA macro array	Extract from seed	The rise in antioxidant gene expression [39]

Antimicrobial	Sequential dilution of plant extracts mixed with 1 ml of DPPH	Methanolic extracts (seeds), dichloromethane, and *n*-hexane	Antioxidant properties by thin-layer chromatography (TLC) qualitative plates test. For the DPPH test, no low activities for methanolic and *n*-hexane extracts were observed, and there was no activity for dichloromethane extract [40]

Anticancer	MTT test	Hydroalcoholic leaves extract	Notable proliferative reduction of A375 and B16 cancer cell lines [41]

Acetylcholinesterase (AChE)	The activity of enzymes evaluated at visible wavelengths	Ethanolic extract, essential oil fraction, decoction, and from aerial portions	No inhibitory observed through the use of the ethanolic extract, and 25% inhibited using 5 mg/ml of plant decoction; 28% of AChE inhibition by 0.1 mg/ml of essential oil [18]

**Table 5:** Pharmacological activities of *M. sylvestris* proclaimed in patents.

Activities	Extract/formulations	Findings
Skin whitening	*M. sylvestris* and other plant extracts	High pigmentation inhibition effect and excellent skin whitening [42]

Anti-inflammatory	Flowers' hydroalcoholic extract and associations	Antiulcer by topical application and anti-inflammatory [7]

**Table 6:** Other related medicinal uses of *M. sylvestris*.

General use	Parts used	Preparation	Specific use
Vaginal disorders	Flowers and leaves	Decoction	Vaginal itching [43]
Pain	Root and leaves	The vapor of decoction (*M. sylvestris* association)	Lumbar ache [44]
Urological disorders	Fruit	Infusion	Irritation of urinary organs, protector of bladder mucous [45]

Respiratory complaints	Fruit	Infusion	Cough [46]
	Aerial parts	Decoction	Respiratory diseases, cough, sore throat, bronchitis [47, 48]
	Leaves/flowers	Leaves/flowers	Pectoral asthma, spasmolytic, expectorant, cough, and emollient [49, 50]
	Whole plant	Infusion	Chronic bladder ulcer, bladder pains [46, 51, 52]

Inflammation	Leaves, flowers, and whole plant	A crushing plant	Rheumatism, the local application against arthritis [53]

Haemorrhoidal	Leaves	Vapour, infusion	Antihaemorrhoidal [54, 55]

Dermatological ailment	Flowers and leaves	Infusion, decoction	Astringent, acne [49, 56]
	Decoction	Emollient	Roots [57]

Menstrual pains	Roots	Decoction	Menstrual pain [58, 59]
	Flowers	Infusion	Dysmenorrhoea [60]

Gastrointestinal disturbance	Whole plant	Decoction	Laxative effects or depurative, against abdominal pains [61]

Other relevant uses	Roots	Decoction	Fever, abortion, weakness, hypertension, and menstrual pain [58, 59, 62]
	Flowers/leaves	Infusion	Soothing, sedative [49, 63]

**Table 7:** Traditional uses of *M. sylvestris*.

Country/region	Used part/s	Use/s (reference)
Iran	Different parts	Cough, expectorant, clear the lung, lubricant, swellings, laxative [64], respiratory diseases of animals, immunomodulation [65]
Pakistan	Leaves	Unspecified method: relaxing activity, gastric mucus, anti-inflammatory, indigestion, diuretic, bladder ulcer [66]
Algeria	Flower	Infusion: antiseptic for the reproductive system, to treat canker sores, colds, constipation, asthma, otitis, colic, abdominal pain, astringent, antiseptic, softening, insect bites, swelling, boils, abscesses [67]
Turkey	Roots	Infusion: abortive [68]
Europe	Aerial parts	Constipation, diarrhea, rumination, tympanism, abdominal colic [69]
Italy	Leaf, root, flower	Leaves decoction or infusion: bronchitis, weight loss, cold, cough, cystitis, belly pain. Crushed leaves: toothache, whitlow [70]
Cyprus	Leaves	Consumed and cooked daily: antidiabetic [71]
India	Aerial parts Different parts of the whole plant	Stimulates the uterus, intestines, ulceration of urinary bladder, cough, enlargement of the spleen, jaundice, sore throat, anti-inflammatory, cooling, mucilaginous [72]. Eaten twice a day to strengthen weak eyesight [73, 74]
Brazil	Unspecified	Infusion: tonsillitis wound, rheumatism, uterine inflammation, boil, diuretic, cleanser [75]
Slovakia	Aerial parts	Food [76]
Syria	Flowers, leaves	Used as a laxative, respiratory infections, cough, mouth wash [77]
Portugal	Unspecified	Unspecified method: treatment of infections [61]
Spain	Aerial parts	Infusion: *Urtica dioica* stings and fever, bruises, wounds, laxative [62, 78], cold, kidney malfunction, dysmenorrhoea, gastralgia [60]
Morocco	Roots, leaves	Urinary or respiratory disorders, cataplasm or decoction [79]
Costa Rica	Whole plant	Unspecified method: ornamental [80]
Poland	Fruits	Eaten raw, immature
Lebanon	Flowers, leaves	Used to treat arthritis and rheumatism [66]

**Table 8:** Major phytochemical compounds detected in the methanolic extract of *M. sylvestris*.

Serial no.	RT (min)	Molecular weight	Exact mass	Chemical structure	MS fragment ions	Pharmacological actions
1.	3.396	333	333.303165	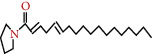	55,81,98,1 13,150,220,264,333	Unknown

2.	3.218	127	127.1360993	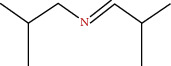	57,84,112	Unknown

3.	3.476	78	78.013936	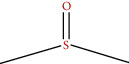	63,78	Anti-inflammatory and antioxidant

4.	3.590	127	127.1360993	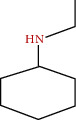	55,71,84,9 8,127	Anti-inflammatory and antioxidant

5.	3.877	141	141.15175	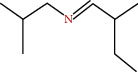	57,69,98,1 13,126	Antistereochemistry

6.	4.306	129	129.115364	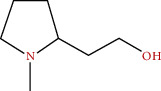	55,84,98,1 29	Unknown

7.	4.449	129	129.115364	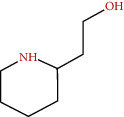	56,84,98,1 28	Antimicrobial, antimalarial, antibacterial

8.	4.563	155	155.167399	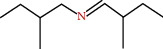	56,70,84,9 8,113,127, 140,154	Antimicrobial activity

9.	4.649	184	184.121178	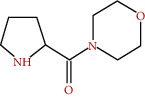	56,70,86,1 14,142	Antimicrobial activity

10.	5.215	191	191.043856	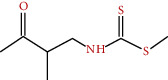	57,85,143, 191	Antibacterial activity

11.	6.057	238	238.068868	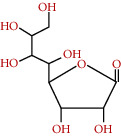	61,73,84,1 12,127,142,159,189,220	Antibacterial activity

12.	7.041	150	150.06808	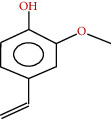	51,77,89, 107,135	Antioxidant, antimicrobial, and anti-inflammatory

13.	8.025	190	190.058971	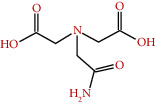	71,101, 127,146, 172,190	Unknown

14.	7.916	207	207.039239	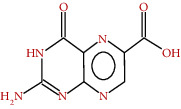	57,69,105, 149,163, 207	Anticancer, antiviral

**Table 9:** Energetic value (Kcal/100 g of dry weight), macronutrients' composition (g/100 g of dry weight), and moisture (g/100 g of fresh weight) of different *M. sylvestris* components [2].

	Leafy flowered	Immature fruits	Flowers	Leaves
Energy	372.43 ± 1.08 b	393.45 ± 4.41 a	372.02 ± 2.13 b	359.72 ± 1.10 c
Reduced sugars	10.46 ± 0.70 b	2.09 ± 0.12 d	13.95 ± 0.16 a	6.22 ± 0.49 c
Ash	10.76 ± 0.04 b	12.83 ± 0.78 a	10.54 ± 0.30 b	13.53 ± 0.11 a
Fat	3.09 ± 0.27 b	8.96 ± 0.22 a	2.84 ± 0.37 b	2.76 ± 0.40 b
Proteins	14.26 ± 0.44 a	3.26 ± 0.25 d	8.50 ± 0.51 c	12.25 ± 1.01 b
Carbohydrates	71.89 ± 0.35 c	74.96 ± 1.10 b	78.12 ± 0.44 a	71.46 ± 0.81 c
Moisture	77.26 ± 1.34 a	45.60 ± 0.97 d	72.49 ± 1.89 c	76.30 ± 0.54 b

**Table 10:** Sugar composition (g/100 g of dry weight) of numerous *M. sylvestris* components (mean ± SD; *n* = 3).

Leafy flowered stems	Immature fruits		Flowers	Leaves
Raffinose	*Nd*	0.26 ± 0.03 a	*Nd*	*Nd*
Trehalose	3.09 ± 0.03 a	*Nd*	1.47 ± 0.06 c	2.67 ± 0.11 b
Sucrose	3.30 ± 0.10 a	0.11 ± 0.03 d	2.47 ± 0.05 c	3.97 ± 0.03 b
Glucose	4.74 ± 0.18 b	1.52 ± 0.07 d	7.36 ± 0.13 a	3.15 ± 0.43 c
Fructose	3.53 ± 0.18 b	0.40 ± 0.03 d	8.72 ± 0.14 a	1.82 ± 0.23 c
Total sugars	14.67 ± 0.49 b	2.30 ± 0.10 d	20.02 ± 0.26 a	11.61 ± 0.51 c

In each row, different letters mean significant differences (*p* < 0.05) [2].

## Data Availability

All the data used to support the findings of this study are included within the article.
